# Meningioma and associated cerebral infarction in three dogs

**DOI:** 10.1186/s12917-020-02388-2

**Published:** 2020-06-05

**Authors:** Lisa Frank, Laura Burigk, Annika Lehmbecker, Peter Wohlsein, Alexandra Schütter, Nina Meyerhoff, Andrea Tipold, Jasmin Nessler

**Affiliations:** 1 Department of Small Animal Medicine and Surgery, University of Veterinary Medicine Foundation, Hannover, Germany; 2 Department of Pathology, University of Veterinary Medicine Foundation, Hannover, Germany

**Keywords:** Meningioma, Infarction, Vascular, Focal ischemia, Canine, Dog

## Abstract

**Background:**

In dogs, meningiomas mostly cause chronic progressive clinical signs due to slow tumor growth.

**Case presentation:**

In contrast, three dogs were presented with the history of chronic generalized tonic-clonic seizures and peracute deterioration with sudden onset of neurological deficits in accordance with an extensive unilateral forebrain lesion. Magnetic resonance imaging examinations of the dogs revealed a well-delineated extraaxial T2W hyperintense mass in the rostral forebrain with homogeneous contrast enhancement. Additionally, an intraaxial, well-demarcated, unilateral lesion was apparent in the parenchyma supplied by the middle cerebral artery. In two cases, necropsy revealed meningothelial meningioma in the rostral fossa and marked eosinophilic neuronal necrosis, a sign of ischemia, focal malacia, edema and gliosis in the temporal lobe and hippocampus because of a focal thrombosis of the middle cerebral artery. In the third case symptomatic treatment resulted in improvement of clinical signs enabling a good quality of life for the patient.

**Conclusions:**

In dogs with structural epilepsy caused by meningioma, acute deterioration of clinical signs can be associated with ischemic infarctions as a potential complication.

## Background

Meningiomas are the most common primary intracranial tumors in dogs [1] and occur more frequently in older dogs [2] and dolichocephalic breeds [3, 4]. Meningiomas arise from the meningothelial cells of the arachnoid cap or pia mater [5] and are located extra-axially, mostly closely located to the calvarium [4] and often involve the olfactory/frontal region [6]. In dogs, meningiomas mostly cause chronic progressive clinical signs due to slow tumor growth [3, 4, 6–8]. On the other hand, ischemic cerebral infarctions are a consequence of end artery closure leading to peracute neurological signs depending on the affected brain region [9, 10]. In 50% of dogs with ischemic infarctions an underlying disease can be diagnosed [11]. In human medicine 25% of primary brain tumors associated with ischemic stroke consist in meningioma [12].

In this report meningioma was diagnosed in three dogs. All three dogs suffered from an unusual acute worsening of neurological signs presumably because of associated ischemic cerebral infarction.

## Case presentation

### Materials and methods

All examinations were performed with written informed owner’s consent according to ethical guidelines of the University of Veterinary Medicine Hannover, Foundation. A resident of the European College of Veterinary Neurology performed the neurological examinations in every dog.

Magnetic resonance imaging (MRI; 3.0 T MRI scanner Achieva, Philips Medical Systems, Best, The Netherlands) and cerebrospinal fluid (CSF) sampling were obtained in general anesthesia (premedication: diazepam 0.5 mg/kg intravenously (i. v.), levomethadone with fenpipramide 0.2 mg/kg i. v. (L-Polamivet®, MSD Tiergesundheit, Unterschleißheim, Germany); induction of anesthesia: propofol dose to effect 1–3 mg/kg i. v.; orotracheally intubation and connection to a semiclosed circle absorber system (Anesthesia ventilator, Cato®; Dräger, Germany); maintenance of anesthesia: isoflurane in an oxygen/air mixture (1:1, flow 50 ml/kg/min)) in all dogs.

If the dogs were euthanized (dog 1 and 2) pentobarbital (100-357 mg/kg, Euthadorm® CP-Pharma Handelsgesellschaft mbH, Burgdorf, Germany) was administered i.v.

Full post mortem examination of the dogs in case 1 and 2 have been done. Representative samples of parenchymal organs, including the whole brain, were fixed in 10% neutrally buffered formalin. The samples were trimmed and embedded in paraffin wax, cut at 3–5 μm and sections were stained with hematoxylin & eosin (HE). For further evaluation of the meningiomas, immunohistochemistry for vimentin (Vimentin, monoclonal mouse, diluted 1:100, Dako, Santa Clara, USA), pan-cytokeratin (AE1/AE3, monoclonal mouse, diluted 1:500, Dako), glial fibrillary acidic protein (GFAP, polyclonal rabbit, diluted 1:1000, Dako) or S-100 protein (S-100, polyclonal rabbit, diluted 1:800, Sigma-Aldrich, St. Louis, USA) using the avidin-biotin-peroxidase complex (ABC) method (Vector Laboratories, Burlingame, USA) was performed. In case 2, additionally immunohistochemistry detecting neuron specific enolase (NSE, monoclonal mouse, diluted 1:100, Dako) and laminin (Laminin, polyclonal rabbit, diluted 1:75, quartett GmbH, Berlin, Germany) were applied [13].

### Dog 1

A client-owned 13-year-old Miniature Schnauzer, male intact, showed non-progressive generalized tonic-clonic seizures since 3 months without obvious interictal abnormalities. The animal was presented to the neurology service of the Department for Small Animal Medicine and Surgery of the University of Veterinary Medicine Hannover, Foundation, because of peracute worsening of clinical signs. The neurological examination revealed an ambulatory tetraparesis with proprioceptive deficits on all four limbs with a left sided accentuation and ongoing oro-facial seizures on the left side of the face. The dog was obtunded and disorientated, showed compulsive pacing and circling to the right. Menace response and vision were absent on both eyes, while pupillary light reflex (PLR) was normal. Nasal nociception was reduced in the left nostril. Other cranial nerves and spinal reflexes were unremarkable. Neuroanatomical localization was a forebrain lesion with a right-sided accentuation.

General examination revealed a mild systolic heart murmur, a small tumor on the left upper eyelid and moderate dental callus.

Blood examination revealed mildly to moderate increased liver enzymes (alanin-amino transferase (ALT) (205 U/l; reference: < 50 U/l), glutamate dehydrogenase (GLDH) (40.2 U/l; reference: < 6 U/l), alkaline phosphatase (ALP) (263 U/l; reference: < 150 U/l)) and urea (65 mg/dl; reference: 20–50 mg/dl). Blood cell count, renal parameters, triglycerides, cholesterol, glucose, electrolytes and thyroid hormones were unremarkable. Non-invasive blood pressure measurements (petMAP, Ramsey Medical, Inc., Tampa, USA) were in the reference range (systolic 120 mmHg, diastolic 64 mmHg). In radiographic examinations of thorax and abdomen multiple spondylarthrosis, hepatomegaly, mineralization in both kidneys and urolithiasis were seen. Renal calculi (up to 1 cm in diameter) and mineralization in both kidneys were also detected during abdominal sonography and furthermore minimal dilatation of the urethra were noted. Urine analysis and microbial testing revealed struvite uroliths and cystitis associated with *Escherichia coli*. No signs of septicemia were present. Heart sonography revealed low-grade mitral valve endocardiosis.

**Fig. 1:**
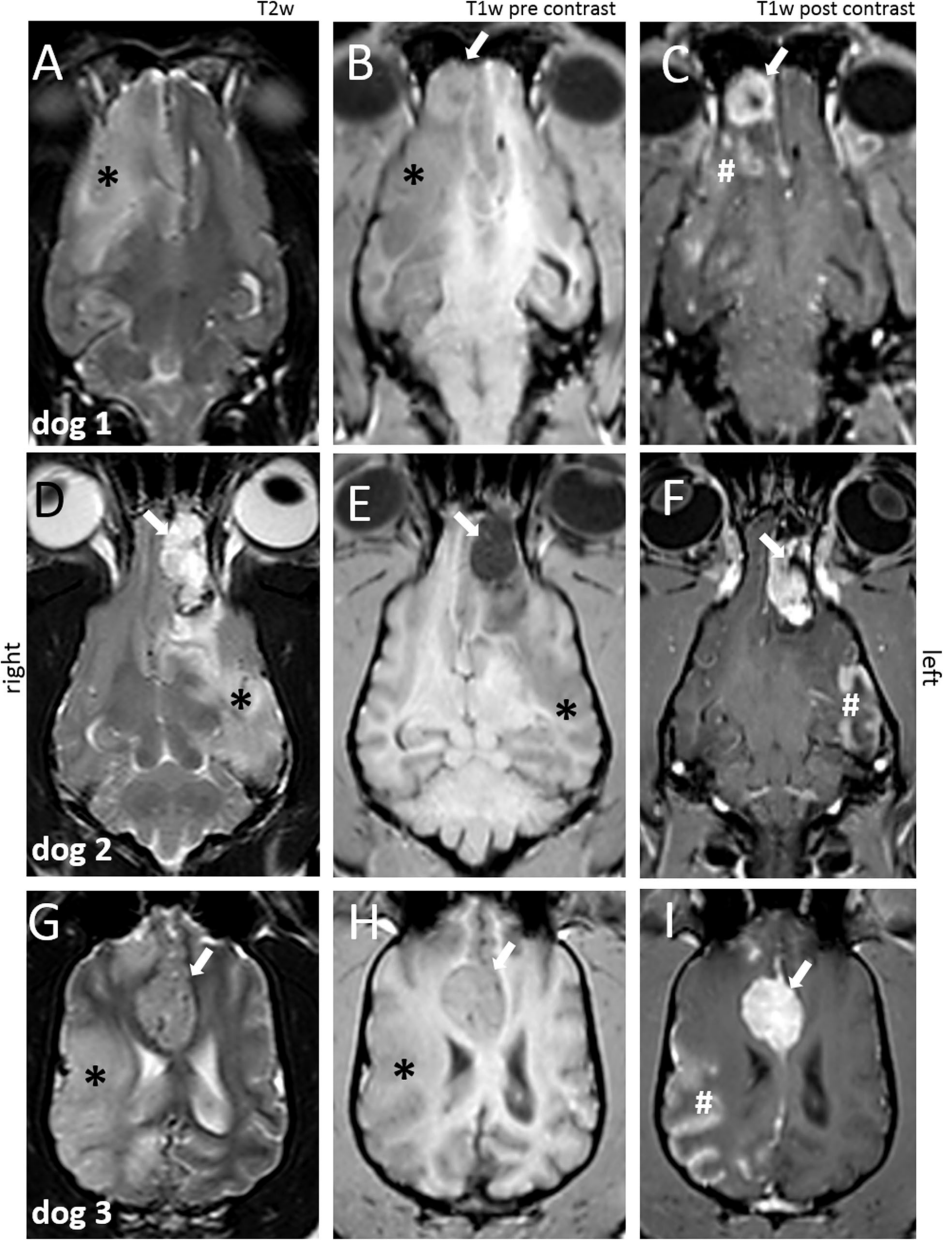
Magnetic resonance imaging (MRI) of the brain. Dorsal view T2 weighted (T2W) (**a**, **d**, **g**), T1W (**b**, **e**, **h**) and T1W post contrast (**c**, **f**, **i**): dog 1: (**a** - **c**), dog 2: (**d** - **f**), dog 3: (**g** – **i**). Well-delineated extraaxial mass (arrow), T1W hypo- to isointense, T2W hyperintense to surrounding tissue, homogeneous contrast enhancement. The mass is compressing the olfactory bulb (B-F)/ frontal lobe (G-I). An additional intraaxial lesion of the parenchyma supplied by the middle cerebral artery, hypointense in T1W and hyperintense in T2W (asterisk) to normal grey matter with heterogeneous ring enhancement (hash)

MRI demonstrates a well-delineated extraaxial mass at the rostral part of the right olfactory bulb, hyperintense in T2weighted (T2W), fluid inversion recovery (FLAIR) and gradient echo (GE) images and isointense to surrounding tissue in T1W sequence. The mass displayed a marked homogeneous contrast enhancement and had a diameter of approximately 1 cm compressing the olfactory bulb (Fig. 1). Additionally, an associated intraaxial, well-demarcated lesion with a much bigger extent was apparent. This lesion included the whole parenchyma supplied by the right middle cerebral artery (MCA) (right occipital, frontal and temporal lobes and lateral part of the olfactory lobe). The second lesion was hyperintense in T2W, FLAIR, and GE and hypointense in T1W images and displayed moderate heterogeneous ring enhancement and mild to moderate mass effect causing a midline shift to the left. Examination of cerebrospinal fluid revealed a disruption of the blood brain barrier with markedly increased protein (177.7 mg/dl; reference: < 30 mg/dl), albumin (111.63 mg/dl; reference: < 20 mg/dl), and a mild increase in leukocytes (28/3 μl; reference: < 9/3 μl; 35% lymphocytes, 28% neutrophilic granulocytes, 33% macrophages, 4% monocytes). Meningioma with peritumoral edema or occlusion of the right MCA was suspected. Differential diagnosis list included lymphoma or histiocytic sarcoma.

Because of the owner’s request the Miniature Schnauzer was humanly euthanized.

**Fig. 2:**
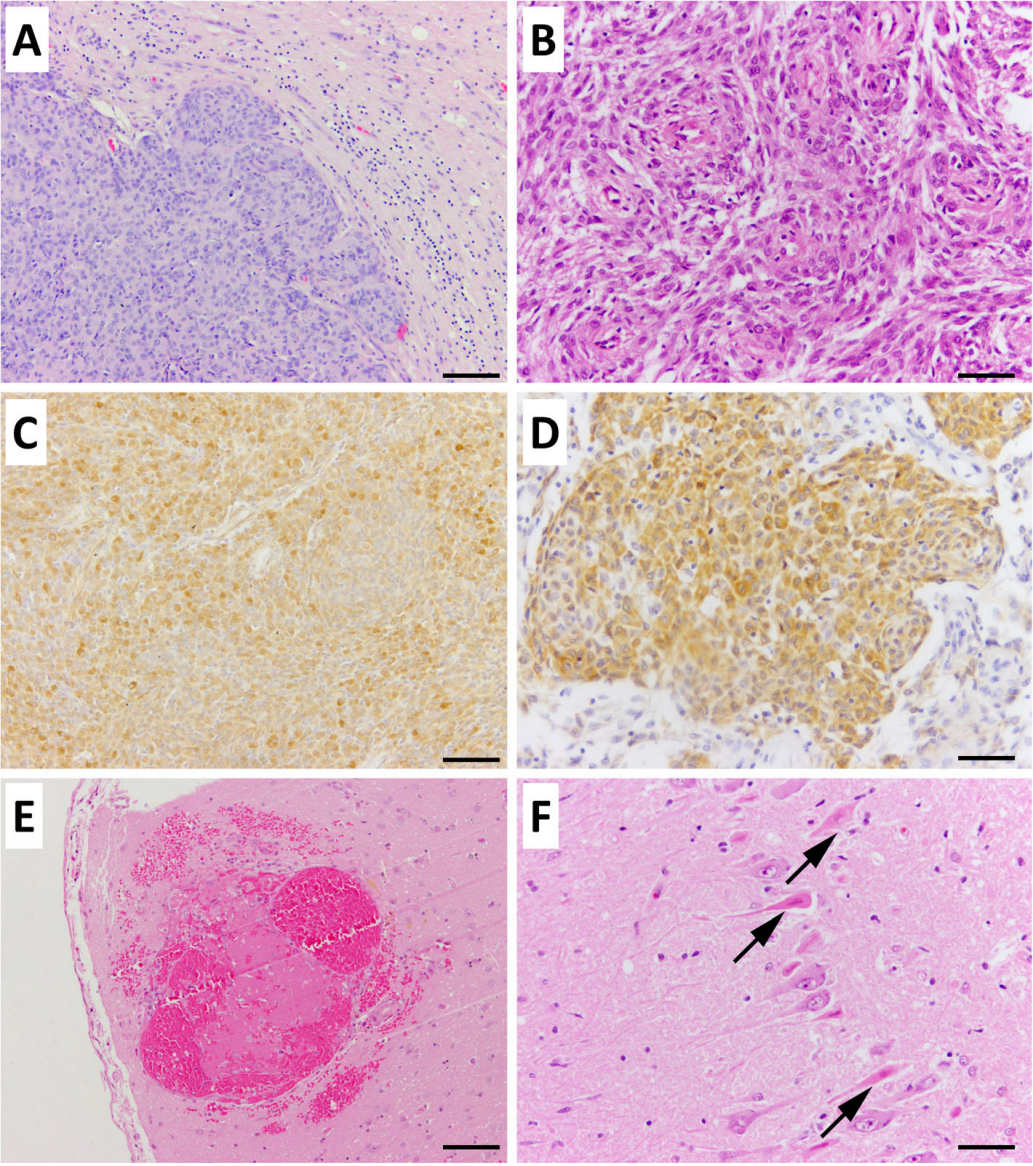
Histopathology. Meningothelial meningioma (dog 1: (**a**, **c**, **e**, **f**), dog 2: (**b**, **d**) – Epitheloid cells, indistinct cell margins, round to oval nuclei. HE, bar 200 μm and 100 μm respectively (**a**, **b**). Immunohistochemical examination: High-intensity, diffuse cytoplasmic staining for vimentin, Bar 200 μm (**c**) and cytokeratin (**d**), Bar 100 μm. Vascular changes represented by a massive thrombus formation within cerebrum (**e**), Bar 100 μm. Neuronal necrosis in the right temporal lobe - Eosinophilic, shrunken, triangular neurons and regressive nuclear changes (arrows). Bar 100 μm (**f**)

Gross examination of the brain revealed a 1.5 × 1 x 1cm^3^ large tumor alongside the right olfactory bulb, adherent to the skull and meninges with a rough and humped texture. A swelling of the right cerebral hemisphere was accompanied by flattening of the gyri and sulci in this area. Histopathologically the mass was identified as meningothelial meningioma (Fig. 2), composed of multiple lobules and whorls of medium-sized, polygonal cells with a moderate amount of eosinophilic cytoplasm and indistinct cell borders. The cells contained a central, medium-sized, round to oval nucleus with finely stippled chromatin and one small nucleolus. The mitotic index was 0 per 10 high power fields (HPF). The tumor showed compression and focal invasion of the adjacent brain parenchyma forming finger-like protrusions. Immunohistochemistry revealed neoplastic cells mildly positive for vimentin and negative for GFAP, pan-cytokeratin and S-100 staining. In the right temporal lobe, a multifocal mild to moderate eosinophilic cortical neuronal necrosis was found, a sign of ischemia. The location of the neuronal necrosis was not directly connected to the meningioma. Isolated vacuoles located in white matter and mild astrogliosis were detected in the left cerebral hemisphere and brainstem. Vessels of the right hemisphere showed moderate to severe swelling of endothelial cells with increased amount of mitotic figures besides hyaline necrosis of vascular walls. A focal thrombus of a small vessel with partly re-endothelialization, hemorrhage and isolated plasma extravasation was visible as well as mild to moderate multifocal, perivascular lympho-histiocytic inflammation of parenchyma and meninges. The presence of endothelial swelling, capillary wall thickening, perivascular lymphocytes, macrophages and plasma cells, gitter cells, microgliosis, spongiosis, hemorrhages, ghost neurons, astrocytosis as well as the absence of neutrophils led to the conclusion that the lesion was at least 10 days old.

These findings confirmed the clinical suspicion of meningioma of the right olfactory bulb histologically classified as meningothelial subtype of grade II and ischemic infarction of the right cerebral hemisphere. Additionally to the changes in the brain, the dog showed focal extrusion of disk material in the area of L1-L2 and multifocal marked ankylosing spondylosis within the spinal column, bilateral multiple leydig cell tumors in the testes, a cortical adenoma within the adrenal gland, a mild multifocal endocardiosis of the heart, mild inflammatory changes within the kidney and the prostate and a 1 cm in diameter urolith within the bladder. All these findings are not considered to be in connection with the meningioma or the vascular changes of the brain.

### Dog 2

An intact female, client-owned 15-year-old Siberian Husky was presented because of oro-facial seizures with secondary generalization since 1 year. The dog was pretreated with phenobarbital 0.9 mg/kg bodyweight twice daily (BID) per os (p. o.) (Phenoleptil®, CP-Pharma Handelsgesellschaft mbH, Burgdorf, Germany) by the referring veterinarian. At the day of presentation, the dog experienced peracute worsening of clinical signs. The dog was in lateral recumbency with reduced consciousness. The body temperature was 40.4 °C, mucous membranes were reddened with a capillary refill time of 2 s and the heart rate accounted 200 beats per minute. At the neurological examination the dogs was ambulatory tetraparetic, with severe ataxia on all limbs and reduced proprioception with a right sided accentuation. She was obtunded and disorientated, showed convulsive pacing, circling to the left, and a decreased menace response of the right eye. The neuroanatomical localization was left forebrain. Secondary to this a neurogenic shock and central hyperthermia was suspected, but at this time point septicemia could not be excluded.

Blood cell count, renal and liver parameters, glucose, electrolytes, thyroid hormones and urine analysis were unremarkable, which made septicemia unlikely. Mild hepatomegaly was evident on abdominal radiographic examination.

MRI demonstrated a well-delineated, extraaxial, focal mass in left olfactory bulb with a size of 3.5 × 1.1 × 1.6 cm^3^. It produced a mild midline shift in the rostral brain region to the right. It appeared heterogeneous hyperintense in T2W, FLAIR, GE, hypointense in T1W with multifocal signal void in all sequences. Marked heterogeneous contrast enhancement was visible. Adjacent to this mass lesion an extended intraaxial lesion of the cortical grey matter and underlying white matter of the left cerebral hemisphere was visible. The area corresponded to brain tissue supplied by the left MCA. The intraaxial lesion was moderately to well-demarcated with a mildly inhomogeneous, hyperintense appearance in T2W, FLAIR, GE sequences and isointense in T1W (Fig. 1). Gyri of the left cerebral hemisphere were swollen and sulci flattened. Mild mass effect shifted the midline to the right. In the cortical grey matter, an indistinct heterogeneous contrast enhancement existed. Cerebrospinal fluid examination was unremarkable. Meningioma with perivascular edema was suspected. Differential diagnoses included round cell tumor, encephalitis, and ischemic infarction. The dog was euthanized on owner’s request.

At neuropathological investigation, the tumor appeared as a focal extraaxial mass at the left ethmoidal fossa with compression of the underlying olfactory bulb. Histopathologically, the mass was diagnosed as meningothelial meningioma (Fig. 2) with a mitotic index of 0 per 10 HPF. The tumor showed no invasive growth into the adjacent brain parenchyma. Immunohistochemical staining showed tumor cells positive for NSE, pan-cytokeratin and S100 and negative for vimentin, GFAP, and laminin. The left cerebral hemisphere was macroscopically swollen and pale. Histopathology showed eosinophilic neuronal necrosis in a laminar pattern in the left olfactory bulb, in the deep cortex of frontal and parietal lobe and area of the left hippocampus with severe astrogliosis and neuronophagia. There was neuropil oedema, astrocytosis, chromatolysis, focal diapedesis of erythrocytes, mild spongiosis whereas endothelial swelling, infiltration of inflammatory cells and gliosis was missing. Taken together, the approximate estimate age of the lesion is probably at least 6 h old.

The final diagnosis was meningothelial meningioma grade I and suspected ischemic infarction of the left MCA. Additionally, a thromboembolic infarction of the left adrenal gland was detected. Within other investigated organs no evidence of hypoxic lesions were evident, however a severe, chronic, focal, granulomatous nephritis, most likely due to passages of parasites, and a hematoma within the spleen were found.

### Dog 3

A client-owned Jack Russel Terrier female intact, 10 years old experienced recurrent generalized tonic-clonic cluster seizures with salivation since 2 months. General examination was unremarkable except a small mass palpable in the right cranial mammary complex. During neurological examination, the dog was in a postictal condition: she was disorientated, agitated and mildly ataxic with mild proprioceptive deficits on all 4 limbs. Menace response was absent in both eyes, with normal PLR. Neuroanatomical localization was a diffuse forebrain lesion. Laboratory parameters were within reference range. Thoracic radiographic examination and abdominal sonography were unremarkable.

**Fig. 3:**
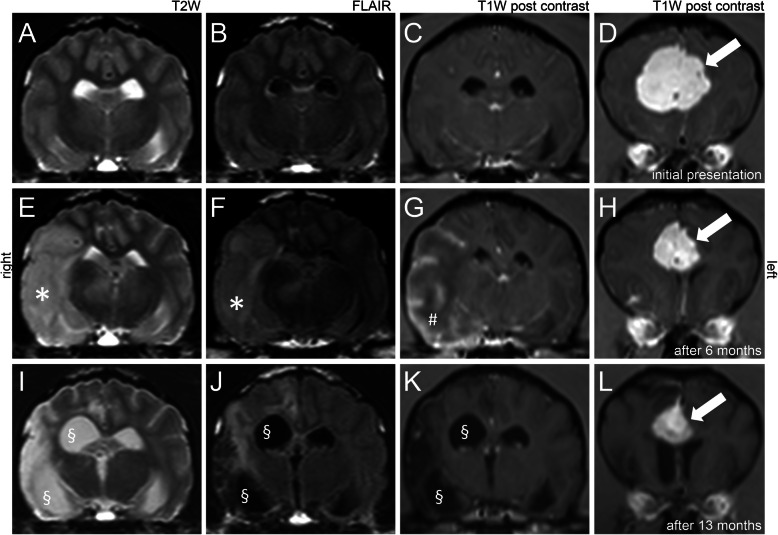
Magnetic resonance imaging (MRI), transversal view of dog 3, follow up examinations. T2weighted (T2W) imaging (**a**, **e**, **i**), Fluid attenuation inversion recovery (FLAIR) (**b**, **f**, **j**), T1W post contrast (**c**, **d**, **g**, **h**, **k**, **l**) on the level of the pituitary gland (**a**-**c**, **e**-**g**, **i**-**k**) and the level of the septal nuclei (**d**, **h**, **l**). MRI performed at initial presentation (**a**-**d**) with only a subfalcine mass present (arrow). MRI immediately after presentation due to peracute onset of clinical signs attributable to a lesion in the right cerebrum (**e**-**h**). The previously reported mass is of decreased size (arrow) but otherwise unchanged. An additional intraaxial lesion attributable to an ischemic infarction of the right middle cerebral artery is visible. It is sharply delineated and involved the right cerebral hemisphere (asterisk). Marked homogeneous contrast enhancement of the outer layers of the cerebral cortex in the affected brain region is apparent (hash). Control MRI 13 months after initial presentation (I-L): the mass is reduced in size (arrow). In the area of the former presumed ischemic infarction, loss of parenchyma causes increased volume of the right lateral ventricle (section)

MRI of the brain revealed a round extraaxial, well-demarcated mass originating from the falx cerebri at the level of the frontal lobes. The mass was inhomogeneous hyperintense in T2W, FLAIR, GE and isointense in T1W. Contrast enhancement was strong and homogeneous with a meningeal tail sign. The mass distorted the lateral ventricles and both frontal lobes. No other changes were visible in initial MRI examination. CSF examination was not performed on owner’s request. Meningioma was suspected (Fig. 1, 3a-d).

Therapy included complete surgical resection of all mammary complexes, application of phenobarbital 2 mg/kg BID (Luminal vet.®, Desitin Arzneimittel GmbH, Hamburg, Germany) and radiotherapy of the brain tumor. Histopathological examination of the mammary tumors characterized them as complex carcinoma and multifocal simple, partly cystic adenomas.

The dog recovered well, showed no neurological signs and no further seizures. Control MRI examination 3 months after radiation revealed that the mass was mildly decreased in size but otherwise unchanged.

Six months after initial presentation the dog was presented in the emergency service due to peracute clinical deterioration. She was disorientated and agitated and showed a right sided head turn. She was non-ambulatory tetraparetic with proprioceptive deficits on all 4 limbs with a left sided accentuation. Her menace response was absent on both eyes with normal PLR and suspected decreased vision. Nociception on the left nostril was decreased. Additionally, continuously focal oro-facial seizures in the left face was observed. General examination and blood pressure measurements were unremarkable. Repeated abdominal sonography stayed unremarkable.

The following MRI displayed the previously reported mass with decreased size but otherwise unchanged. However, an additional intraaxial lesion was visible (Fig. 3e-h). It was sharply delineated and involved the temporal, parietal and occipital lobe of the right cerebral hemisphere in the region supplied by the right MCA. It appeared hyperintense in T2W, FLAIR, GE and diffusion-weighted imaging. The apparent diffusion coefficient was hypointense compared to T2W. Marked homogeneous contrast enhancement of the outer layers of the cerebral cortex in the affected brain region was apparent. Owners declined CSF examination. Beyond the already clinically established diagnosis of a presumed meningioma, an additional territorial ischemic infarction of the right MCA was suspected.

Subsequent symptomatic therapy included additional antiepileptic medication (Clonazepam 0.5 mg/kg p.o. TID (Rivotril®, Roche Pharma AG, Grenzach-Wyhlen, Germany), Levetiracetam 20 mg/kg p.o. TID (SanacorpLangenhagen, Germany)), hand feeding, and continuous rate infusion. After six days the dog’s clinical signs improved and she started to walk again with waxing and waning consciousness, compulsive circling to the right and decreased vision. She could be discharged from hospital at day nine after onset of the tetraparesis. At this time point she was alert, ambulatory and could follow commands. Only a visual deficit of the left eye with reduced menace response and normal PLR was noticeable. Further follow up information are available until time of writing (1.5 years after first presentation): The dog experienced generalized tonic-clonic or focal oro-facial seizures every 2–8 weeks despite treatment with phenobarbital 3.6 mg/kg BID p.o. and levetiracetam 20 mg/kg TID p.o. A control MRI 13 months after initial presentation showed multifocal cavitation of the right cerebral cortex, increased volume of the lateral ventricle due to loss of parenchyma and multifocal, T2W and FLAIR hyperintense intraaxial lesions in the right cerebral cortex without mass effect and without pathological contrast enhancement. These lesions were located at the location of the presumed infarction (Fig. 3i-l). The suspected meningioma was reduced in size in comparison to MRI examinations but otherwise unchanged. According to the owners, the dog’s disability is not recognizable in everyday life despite seizures and she has a good quality of life. During the last follow up visit at the hospital 20 months after initial presentation, the dog was bright and alert and her gait was normal. She showed a mild head turn to the right, intermittent compulsive pacing in circles to the right, and left sided vision and proprioceptive deficits.

## Discussion and conclusions

Meningioma are the most common primary intracranial tumors in dogs [1] and mostly cause chronic progressive clinical signs due to slow tumor growth and benign biological behavior [3, 8]. They mainly arise from arachnoid cap cells covering arachnoid granulations [5], mostly at the point where they project into the dural venous system [14]. On base of the cellular composition and arrangement, various subtypes of meningiomas can be differentiated histologically [15]. The meningothelial pattern is characterized by syncytial whorl-like formations of epithelioid cells with abundant and homogeneous cytoplasm without defined borders. The fibroplastic pattern shows a number of spindle shaped cells creating intersecting streams or bundles supported by dense collagen fibres. In addition, a mixed typed (transitional meninigioma) is reported. If whorl formation with a core of central hyalinization, necrosis and mineralization is found, the psammomatous pattern is present [15]. Especially tumors of the rostral forebrain often do not even cause any obvious neurological signs other than seizures [16]. Progression of clinical signs mostly occurs insidious [3] but as intracranial pressure raises, and intracranial compliance is exhausted, acute deterioration due the caudal cerebellar herniation might occur and present as bilateral brainstem-signs [1]. On the other hand, ischemic stroke is mostly associated with sudden onset of neurological signs indicative of a focal lesion [17]. Multiple causes for occlusion of an intracranial vessel exist: occlusion of the vessel may occur due to emboli originating from a distant part of the body like parasitic, neoplastic or septic emboli or due to thrombemboli arising because of a hypercoagulable state [18]. Thrombi may develop locally if the vessel wall is damaged and uncontrolled intravascular coagulation emerges [19]. Additionally, arteriosclerosis, vasospasm or an external compression of the vessel could lead to narrowing of the vascular diameter and therefor leading to an ischemic infarction [19, 20]. The current case series include two dogs with chronic seizures due to histopathologically confirmed meningothelial meningioma and an unusual peracute onset of unilateral neurological signs. In addition to the meningioma an extensive, well demarcated area of neuronal necrosis in the cortex of one cerebral hemisphere on the side of the meningioma indicates infarction of the ipsilateral MCA, which most probably induced sudden deterioration. In the differential diagnosis acute neuronal cell death caused by excitotoxicity due to prolonged seizure activity should be considered [21]. In contrast to the presented cases, excitotoxicity following seizures causes neuronal necrosis preferred bilaterally symmetrical in specific brain regions as internal portion of the cerebral frontal lobe, hippocampus or cingulate gyri [21].

In the third case post mortem examination was not performed, since the dog recovered well and is still alive at the time of writing. Clinical and MRI features resembled the other two confirmed cases and the MRI diagnosis reinforces the suspected diagnosis of ischemic infarction of the MCA additional to a preexisting meningioma. In human medicine, ischemic infarctions in patients with brain tumors are a rare but well recognized phenomenon [12]. Several hypotheses exist to explain the concurrent existence of brain tumors and stroke: In several reports, the tumor itself or the peritumoral edema compressed or invaded an artery and therefor lead to occlusion of the vessel [20, 22, 23]. Most frequently, infarction is associated with radiation-induced vasculopathy due to radiotherapy [12, 24]. However, brain tumors itself are also capable to affect blood coagulation: Focal or systemic damage of endothelial cells by neoplasia can activate hemostasis [12]. Production of plasminogen activators especially in meningiomas leads to a hypercoagulable state and increases the risk of thrombus formation [25]. In addition, several brain regions are known to actively influence the serum levels of factor VIII activity [26]. A decreased activity of the hypothalamic area or the ventral hippocampus due to damage by the tumor may also induce a hypercoagulable state [25]. Histologically investigated meningiomas of the present Case 1 and case 2 were histologically classified as meningothelial of grade II and grade I, respectively, consistent with the human classification and grading of meningiomas. However, precise correlation of clinical and prognostic data is not as extensively studied in veterinary medicine as in human medicine.

In the presented cases, an intensive work up was performed to exclude systemic causes for ischemic stroke as far as possible. In case one septic emboli due to bacterial cystitis might be a possible reason for stroke, but necropsy could not detect bacteria within the histopathologically diagnosed thrombus. In the third case, radiation-induced vasculopathy could have caused occurrence of stroke, but as radiation was not performed in the author’s clinic, radiation protocol was not available. In the second case, no underlying pathology could be found, but due to adrenal infarction a connection between similar changes in the brain could not be excluded. Interestingly, in all three described cases a very similar distribution of the meningioma and the affected vessel was seen: the tumor was located in the rostral forebrain and the occlusion occurred in the ipsilateral MCA. A direct obstruction of the vessel via compression by the tumor itself is unlikely due to its anatomic localization. However, the mass effect and an additional peritumoral edema increasing the intracranial pressure, could have caused the stroke by indirect compression of the MCA.

In veterinary medicine, to the author’s knowledge this is the first case series reporting a concurrent presence of meningioma and cerebral infarction. An etiological association is feasible. In dogs with sudden deterioration of clinical signs and diagnostic imaging with suspicion of an intracranial infarction despite an extraaxial mass supportive treatment attempt might be worthwhile as demonstrated in case 3. Prognosis seems to be fair regarding the acutely occurring clinical signs caused by the infarction.

## Data Availability

The datasets used and analysed during the current study are available from the corresponding author on reasonable request.

## Abbreviations

BID: Twice a day (lat. “*bis in die*”)
cm: centimeter
FLAIR: Fluid attenuated inversion recovery
GE: Gradient echo
HPF: High power field
i.v.: intravenously
kg: kilogram
l: liter
MCA: Middle cerebral artery
mg: milligram
ml: milliliter
mmHg: millimeters of mercury
MRI: Magnetic resonance imaging
PLR: Pupillary light reflex
p.o.: orally (lat. “per os”)
T1W: T1-weighted
T2W: T2-weighted
TID: Three times a day (lat. “*ter in die*”)
U: Units
